# Commentary: Perceptions of Clinical Dental Students Toward Online Education During the COVID-19 Crisis: An Egyptian Multicenter Cross-Sectional Survey

**DOI:** 10.3389/fpsyg.2022.925434

**Published:** 2022-06-13

**Authors:** Fatima-Ezzahra Faridi, Ghita El Basraoui, Jihad Diouchi, Sonia Ghoul

**Affiliations:** BioMed Unit, International Faculty of Dental Medicine, College of Health Sciences, International University of Rabat, Rabat, Morocco

**Keywords:** COVID-19, education, dental, graduate, students, distance

It is with great interest that we have read the article entitled “*Perceptions of Clinical Dental Students Toward Online Education During the Covid-19 Crisis: An Egyptian Multicenter Cross-Sectional Survey*,” published in the journal “Frontiers in Psychology” (Hassan et al., 2022). We found the article of highly importance considering that (i) little is known about the experience of African countries on remote education during the COVID-19 pandemic and (ii) dental students have been particularly deeply affected by the pandemic not only because of the rapid switch to online education, but also because of the limited practical approaches with patients during this period. Therefore, clinical dental students did not have enough opportunities to practice on patients during the pandemic and it was important to evaluate their perception regarding their training with the new pedagogical approaches set up rapidly to face the pandemic risks and the lack of clinical activities.

The objective of the study was to determine the effects of the COVID-19 pandemic on the perceptions of Egyptian dental clinical students toward online education. Thus, an online survey was conducted among four Egyptian universities, involving two public and two private establishments with the same teaching methods. To achieve this, authors estimated the intended sample size for this study at 235 but did not specify the total number of clinical dental students registered at the targeted universities. For the power analysis parameter of the study, authors referred to Varvara's paper where the response rate was about 85% (Varvara et al., 2021), but they missed to mention the response rate of their own study. Otherwise, authors used Fisher's exact test for the statistical analysis, suggesting the studied samples were small. Obviously, it would have been useful to specify how many dental institutions exist in Egypt and how many dental clinical students were targeted in this study to better appreciate the representativeness of the sample. In Turkey for example, 65 dental schools currently exist, involving 13 private and 52 state universities (Sarialioglu Gungor et al., 2021), while in Tunisia there is only one public dental faculty for the country. The total number of dental faculties in a country should be mentioned to better evaluate the study.

Moreover, the presentation of the dental curriculum in Egypt would have been appreciated to better understand why the study included dental students of 3rd, 4^th^, and 5th year. In fact, clinical activities for students may also vary among countries. In Morocco for example, dental clinical activities begin at the 4th year and not at the 3rd year. These activities continue at the 5th year and become exclusive at the 6th year. Overall, it seems interesting to evaluate the perception of the dental students regarding their training in developing countries as demonstrated by Hattar et al. who showed that in Jordany for example 87% of dental students perceived that the most negatively affected experience during the pandemic was their clinical training (Hattar et al., 2021).

The COVID-19 pandemic had an important impact on the clinical dental student perception of their practices worldwide. Similar studies are welcomed from developing countries to better evaluate the perception of dental students regarding the remote education and limited practical activities during the COVID-19 crisis.

## Author Contributions

GE selected the article. All authors critically read the paper, contributed to the final manuscript, and approved the submitted version.

## Conflict of Interest

The authors declare that the research was conducted in the absence of any commercial or financial relationships that could be construed as a potential conflict of interest.

## Publisher's Note

All claims expressed in this article are solely those of the authors and do not necessarily represent those of their affiliated organizations, or those of the publisher, the editors and the reviewers. Any product that may be evaluated in this article, or claim that may be made by its manufacturer, is not guaranteed or endorsed by the publisher.
